# The Hodgkin Lymphoma Immune Microenvironment: Turning Bad News into Good

**DOI:** 10.3390/cancers14051360

**Published:** 2022-03-07

**Authors:** Victoria Menéndez, José L. Solórzano, Sara Fernández, Carlos Montalbán, Juan F. García

**Affiliations:** 1 Translational Research, MD Anderson Cancer Center Madrid, 28033 Madrid, Spain; victoria.menendez@fundacionmdanderson.es (V.M.); sara.fernandez@mdanderson.es (S.F.); 2 Pathology Department, MD Anderson Cancer Center Madrid, 28033 Madrid, Spain; jlsolorzano@mdanderson.es; 3 Hematology Department, MD Anderson Cancer Center Madrid, 28033 Madrid, Spain; cmontalban@mdanderson.es; 4 Centro de Investigación Biomédica en Red de Cáncer (CIBERONC), 28029 Madrid, Spain

**Keywords:** classic Hodgkin lymphoma, tumor microenvironment, immune cell phenotypes

## Abstract

**Simple Summary:**

Classic Hodgkin lymphoma (cHL) is one of the most enigmatic study models of the tumor microenvironment (TME), in which the Hodgkin–Reed Sternberg (HRS) cells are distributed throughout an abundant but ineffective immune ecosystem. The hyperactivation of HRS cells due to somatic mutations leads to complex interactions with the different subsets of immune cell populations, modeling the pathophysiology of the disease. There are remaining loose ends regarding the identification of the immune functional states in the cHL microenvironment and their influence on tumor cell survival. Here, we review the most relevant immune populations identified in the cHL context, focusing on integrative functional signatures.

**Abstract:**

The classic Hodgkin lymphoma (cHL) tumor microenvironment (TME) is by far the most abundant component of tumors and is responsible for most of their biological and clinical characteristics. Recent advances in our knowledge of these networks in cellular interactions allow us to understand that the neoplastic Hodgkin and Reed Sternberg (HRS) cells, although they are in the minority, are the main architects of this dysregulated immune milieu. Here, we review the major changes that have happened in recent years: from TME as a helpless bystander, reflecting an ineffective immune response, to a dynamic tumor-promoting and immunosuppressive element. The HRS cells promote survival through interconnected intrinsic and extrinsic alterations, boosting pro-tumoral signaling pathways through genetic aberrations and autocrine growth signals, in parallel with abnormal cytokine secretion for the recruitment and selection of the best cell partners for this immunosuppressive TME. In turn, cHL is already proving to be the perfect model with which to address an immune checkpoint blockade. Preliminary data demonstrate the utility of druggable key signaling pathways in this ensemble, such as JAK-STAT, NF-κB, and others. In addition, myriad biomarkers predicting a response await validation by new in situ multiplex analytical methods, single-cell gene expression, and other techniques. Together, these components will define the functional phenotypes with which we will elucidate the molecular pathogenesis of the disease and improve the survival of patients who are refractory to conventional therapies.

## 1. Introduction

The tumor microenvironment (TME) is a crucial determinant of tumor growth, progression, and resistance to chemotherapy in cancer, and classic Hodgkin lymphoma (cHL) is one of the most representative examples [1]. cHL tumors are characterized by a minor B cell-derived monoclonal proliferation of Hodgkin and Reed-Sternberg (HRS) cells diluted in an abundant inflammatory microenvironment, which is the main component of the tumor mass [2].

Observational data favor the general idea that genomic lesions occurring early in the neoplastic HRS clone lead to aberrant activation of signaling pathways and transcription factors [3,4,5,6], which induces specific secretory profiles of growth factors, chemokines, and cytokines that, together, foster and shape the microenvironment [6,7]. This complex process includes remodeling the stroma and cellular matrix, as well as migration and activation of leukocytes [8].

Given that HRS cells are scarce and not prognostically relevant [5], the evidence is strong that the determinants of the cHL therapeutic response are elements of the TME, which features a broad range of known distinct cell types, and many others that have not yet been exhaustively identified [9,10]. In agreement, there are no prognostic differences between nodular sclerosis (NS) subtypes I and II [11]. Tumor cells can release specific molecules or extracellular vesicles to communicate and reshape the TME, and these favor communication with remote regions [12]. At the same time, HRS cell proliferation and apoptotic evasion are supported by interactions with their surroundings in pediatric cHL [9], where the predominant populations are mostly CD4^+^ T cells, and, to a lesser extent, CD8^+^ T cells, B cells, macrophages, eosinophils, plasma cells, mast cells, neutrophils [9,10], dendritic cells (DCs), fibroblasts, and natural killer (NK) cells [2,10].

Previous findings regarding the prognostic and predictive role of the TME in cHL have shown that distinct lymphoid and monocytic compartments could play critical roles [13]. For instance, intratumorally activated cytotoxic T cells are historically associated with worse clinical outcomes [7,9,14], whereas T regulatory cells (Tregs) are correlated with better prognosis [2]. Discrepant findings related to the involvement of macrophage infiltration in the TME suggest that many of our current concepts about monocyte polarization into M1-like and M2-like phenotypes are extremely oversimplified and ignore most aspects of cell plasticity [15,16]. The same occurs with NK cells, neutrophils, myeloid cells, and dendritic cells. Additionally, many other related cell populations play pivotal roles, such as some subsets of T helper (Th) cells, monocytes [13], myeloid DCs (mDC), and plasmacytoid DCs (pDC) [17]. The network of interactions of all these subpopulations in the cellular crosstalk of cHL has not yet been explored.

Forthcoming advances in our knowledge of the cHL TME may identify integrative immune signatures or phenotypes, allowing immunosuppressive and tumor-promoting cell populations to be distinguished from anti-tumor cell types. Finding imbalances in the polarization or the differentiation of these populations rather than different cell frequencies between healthy donors and patients, or between favorable and unfavorable cases, would offer a new way of understanding the biology of cHL and of developing new therapies [18,19].

## 2. T Cells

T cells are the most abundant cell type in the cHL TME [20]. HRS cells release chemokines to attract T cells or some of their subsets, while T cells increase the clonogenic growth of tumor cells and attract more cell populations by releasing other factors [20,21]. This vital relationship is physically manifested by the formation of HRS-T cell aggregates, called rosettes, which are considered to be one of the hallmarks of cHL [22], in which the reactive T cell compartment is remarkably heterogeneous [3] and entirely lacking in senescence markers [23].

CD4^+^ T cells produce cytokines and chemokines with regulatory or inflammatory functions when they detect antigens in the context of the major histocompatibility complex class II (MHC-II) molecules. Alternatively, CD8^+^ T cells use the MHC-I complex to detect antigens and directly kill neoplastic or virus-infected cells. Secondly, T cells can be classified by their differentiation stages, including naive T cells, which have never been exposed to an antigen, central memory (CM) T cells, effector memory (EM) T cells, and terminally differentiated (TEMRA) T cells [3].

Finally, T cells can be grouped by their type of effector response [24]. For instance, type 1 effector responses mediate immunity to many microorganisms [5]. They include Th1 and Th17 cells, cytotoxic T lymphocytes (CTLs), group 1 and group 3 innate lymphoid cells, and some antibody classes [25]. By contrast, type 2 effector responses release cytokines arising from antigen-stimulated CD4^+^ T cell subsets such as Th2 and Th9 cells, antigen-stimulated basophils, and cytokine-stimulated innate lymphoid cells. These cytokines recruit some innate cell populations such as eosinophils, basophils, and mast cells, and enhance the proliferation and differentiation of CD4^+^ T cells [26]. It is notable that T cell subsets are extremely plastic, making intermediate and mixed phenotypes possible. As an example, Th2 cells can co-express typical cytokines from Th1, Th2, and Th9 subsets [24].

## 3. CD4^+^ T Cells

Six CD4^+^ T cell subsets have been described, including Th1, Th2, Th17, T follicular helper (Tfh), Treg, and Th9 cells [24], although the diversity is growing over time. The T cells that make up the HRS rosettes are specifically CD4^+^ T cells [20,27,28], which are a mixed population of Th cells and Tregs [27]. These CD4^+^ T cells express molecules such as *CD40L* [21], *CD80*, and *CD54* [12], which can promote tumor growth and disease progression through the activation of important pathways such as NF-kB [21].

High levels of infiltrating and active CD4^+^ T cells and CD4^+^ T-bet^+^ cells are associated with a better outcome in cHL patients [23,27,29]. However, some authors consider there to be no difference in the total CD4^+^ T cell percentages between cHL patients and healthy donors, although there are differences in T differentiation levels. Specifically, there are more CD4^+^ TEMRA cells and fewer CD4^+^ CM in cHL biopsies [30]. CD4^+^ T cells may be correlated with a favorable outcome due to their possible direct function as cytotoxic effectors, given the frequent MHC-I loss in this tumor [29]. Accordingly, T-bet promotes the differentiation and activation of cytotoxic CD4^+^ T cells, which are enriched in groups with more differentiated T cells [31], supporting the hypothesis.

### 3.1. Th1 Cells

CD4^+^ T helper 1 (Th1) cells produce cytokines such as IFN-γ, IL2, and LT-b [32], favor cytotoxic CD8^+^ T cell activation, and polarize monocytes and macrophages to the M1-like phenotype [27]. They are among the elements of type 1 immunity, along with IFN-γ and IFN-γ-stimulated macrophages, which collaborate to maintain tumor immune surveillance and to reduce tumor growth [25]. In cHL, the CD4^+^ T cells of the TME are mainly polarized to Th1 effectors [3,23,28,29].

A recent study of 47 cHL patients by Dehghani et al. demonstrated that the frequency of Th1 cells (CD4^+^ IFN-γ^+^ IL4^−^) is positively correlated with relapse compared with newly diagnosed patients in peripheral blood [33]. Accordingly, T-bet^+^ Th1 cells [23], EM Th1 (CCR7^−^ CD45RO^+^ EOMES^low^) cells, and Th1 Treg (CD25^+^ FOXP3^+^ T-bet^+^) cells are the CD4^+^ T cell subsets most frequently expanded in cHL tissues compared with healthy reactive lymph nodes (RLNs) [3,30]. However, the total number of Th1 cells is not associated with outcome [33], possibly because the difference does not rely on the number of Th1 cells but on their phenotype, which is probably exhausted and has limited anti-tumor abilities [3,30].

### 3.2. Th2 Cells

Type 2 immunity is involved in many processes, such as anthelmintic immunity, metabolic homeostasis, wound repair, tissue regeneration [25], macrophage polarization towards the M2-like phenotype [27], and differentiation of CD4^+^ T helper 2 (Th2) cells. Classic type 2 cytokines include IL4, IL5, IL9, and IL13 [34], which are responsible for the recruitment of eosinophils (IL5), basophils (IL4), and mast cells (IL4, IL9, and IL13). Th2 and regulatory T cells are attracted by HRS cells through CCL5, CCL17, and CCL22 chemokines [5,20]. Furthermore, IL13 is produced by T and HRS cells to induce Th2 differentiation [20].

High frequencies of Th2 cells are correlated with significantly improved disease-free survival (DFS) and event-free survival (EFS) in cHL [35]. Accordingly, CD4^+^ IFN-γ^−^ IL4^+^ cells (Th2) are significantly more abundant in the peripheral blood of patients in the remission phase compared with newly diagnosed patients. However, some studies have found no association between the number of Th2 cells and prognosis, while relapsed cHL patients have significantly fewer Th2 cells [33].

Additionally, the presence of PD-1^+^ Th2 cells is correlated with worse outcomes and shorter survival in cHL [36]. This is intriguing because less differentiated and less polarized Th2 cells have lower levels of *PD-1* expression in cHL samples compared with RLNs, as happens with Th1 cells [30]. These patterns also suggest that the TME of cHL may switch Th2 towards a less differentiated or exhausted phenotype.

### 3.3. Th17 Cells

Th17 cells produce inflammatory cytokines such as IL17A and IL17F to trigger an inflammatory response [37,38]. Despite mediating anti-tumor responses through recruiting immune cells into tumors, activating effector CD8^+^ T cells, and promoting Th1 polarization, Th17 cells activate angiogenesis and immunosuppressive activities, leading to tumor progression in some models [38]. In cHL, HRS cells release cytokines such as IL6, IL21, IL23, and soluble CD30 to polarize T cells towards a Th17 phenotype. In turn, Th17 cells promote the recruitment of myeloid cells and amplify the inflammatory infiltrate [3].

Th17 cells are related to poor clinical outcomes in B cell lymphoma and diffuse large B cell lymphoma (DLBCL) [39]. In accordance, Th17 cells are enriched in Epstein Bar Virus (EBV)-negative cHL patients compared with healthy individuals [3,28]. Furthermore, survival rate is positively correlated with the Treg/Th17 ratio, suggesting that a higher frequency of Th17 cells may increase disease aggressiveness. However, the correlation between the Th17 infiltrate in cHL with outcome has not been established beyond doubt because the studies employed a variety of technical approaches and biomarkers. Another possible explanation for the discrepancies stems from the different phenotypes that Th17 cells can acquire, since these may have different roles in promoting or suppressing tumor growth. For instance, CXCR5^+^ and CXCR5^−^ CM Th17 cell subsets and CD161^+^ CCR4^+^ Th17 cells are less abundant in primary cHL samples than in RLNs [3,30]. Moreover, almost half of the cHL cases display IL17^+^ and FOXP3^+^ IL17^+^ T cells, which are mainly located in the periphery of HRS cells [3,40]. It is of particular interest that the latter cell cluster has an immunosuppressive capacity and represents an intermediate differentiation stage between Th17 cells and regulatory T cells. The cHL TME may influence Tregs to produce IL17 and to acquire a more immunosuppressive phenotype by releasing TGF-β or other molecules, which are known to promote IL17^+^ Tregs [41]. Even the HRS cells themselves produce TGF-β [21], which is consistent with the hypothesis.

### 3.4. Regulatory T Cells

Tregs are a specialized subgroup of CD4^+^ T cells that are identified either as CD4^+^ CD25^+^ [42] or as CD4^+^ CD25^+^ FOXP3^+^ cells, and whose main functions are to regulate immune responses by suppressing the activation of other CD4^+^ T cell subsets [20,37], and to preserve self-tolerance. However, they also promote tumor progression through a variety of molecules such as CTLA4, IL2, and IL10 [16].

Lymphoma-infiltrating Tregs can suppress the effector function of CTLs in vitro and the activation and differentiation of normal B cells in vivo [42]. HRS cells immune escape is facilitated by the protective shield conferred by CD4^+^ cells rosetting around tumor cells and by polarization towards Tregs [43]. Some researchers have found that high levels of Tregs in cHL tissues are not associated with an adverse prognosis [21], whereas others have found fewer Tregs in relapsed patients [14,37] and a positive association between them and survival [2,5,23,37,42]. All these findings are counter to what has been seen in other tumors [27], in which Treg depletion leads to tumor regression [2]. Additionally, some Treg subsets, such as FOXP3^+^ GrB^+^ cells, are reported to be correlated with improved survival in cHL [5,14], suggesting that some Treg subsets work together with CTLs to reduce tumor proliferation.

### 3.5. Tr1 Cells

Apart from conventional Tregs, cHL-infiltrating lymphocytes contain a large population, named regulatory T 1 (Tr1) cells or induced Tregs, that secrete IL10 and are largely absent from healthy RLNs. It is a strongly immunosuppressive cHL-associated subset of T cells with a high level of expression of the *LAG3* receptor and located in the direct vicinity of *MHC-II*-deficient tumor cells [7,28]. HRS cells produce molecules such as Galectin and EBV-derived LMP1 to induce Tr1 cells [20], that specifically inhibit Th1 effector cells [21]. Interestingly, MHC-II deficiency, which is one of the major ligands of LAG3, is a predictor of unfavorable outcomes after PD-1 blockade in cHL. Although there are not currently enough data to demonstrate the value of LAG3^+^ T cells as a predictor of unfavorable responses, ongoing lymphoma clinical trials are targeting LAG3, including in cHL patients [28,44]. If the therapy against LAG3 is successful, it will confirm that the Tr1 population does contribute to immunosuppression and tumor development in cHL.

### 3.6. T Follicular Helper Cells

Tfh cells are another Th subset located in B cell follicles [34]. They are essential for germinal center formation, affinity maturation, and the development of most high-affinity antibodies and memory B cells [45]. Tfh cells produce Th1-, Th2-, and Th17-associated cytokines, probably because they are potentially derived from these lineages. It suggests that Tfh cells are extremely plastic, as is the case for other T cell subsets. Another singular quality of Tfh cells is based on the stable expression of a high level of *CXCR5*, which is necessary for Tfh cells to migrate to *CXCL13*-expressing B cell follicles [46].

CD4^+^ Tfh cells occur at a significantly lower frequency in cHL than in normal RLNs [30]. This is the converse of what may occur in other hematological malignancies, whereby Tfh cell ratios are higher in patients than in healthy subjects, and lower in treatment-effective patients [47]. Still, Tfh cells have not been closely studied in cHL and their phenotypes have not been clearly established.

## 4. CD8^+^ T Cells

High frequencies of general CD8^+^ T cells in the cHL TME are associated with a better outcome, especially in the advanced-disease group [9,27], whereas worse prognosis is associated with a greater abundance of activated (GrB^+^ TIA1^+^) CD8^+^ T cells [5,7,14,36]. Accordingly, greater numbers of activated CTLs in gene expression profiling and tissue biopsies of cHL patients are associated with unfavorable clinical outcomes [5]. In contrast, high levels of *T-bet* expression are associated with superior disease-specific survival (DSS) [23], suggesting that T CD8^+^ T-bet^+^ cells could be a newly identified subset of exhausted or immunodeficient T CD8^+^ cells that allow tumor progression in cHL. They are linked to other pathological conditions such as autoimmune diseases [48,49].

**Figure 1:**
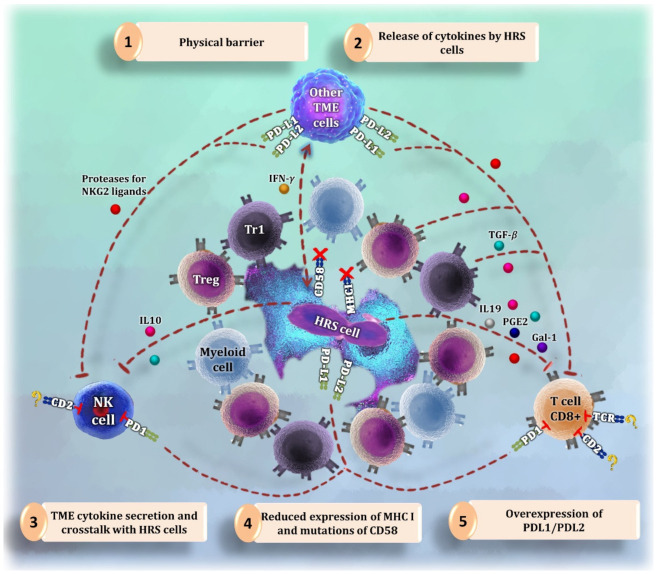
Summary of the different strategies to evade cytotoxic attack by the Hodgkin-Reed Sternberg (HRS cells). (**1**) CD4^+^ T cells and myeloid cells are abundant in the proximity of HRS cells, forming a physical barrier that makes it difficult for CD8^+^ T cells and natural killer (NK) cells to reach tumor cells. (**2**) HRS cells release cytokines and other factors to reduce the activity of cytotoxic cells and to communicate with the tumor microenvironment (TME). (**3**) Following orders from the released signals by tumor cells, macrophages and other microenvironment cells start producing more immunosuppressive molecules and receptors that inhibit CD8^+^ T cells and NK cells. (**4**) One of the mechanisms for cytotoxic cells to become activated is to bind their CD2 receptor with CD58. HRS cells express less *CD58* or have *CD58* mutations to avoid the interaction with CD2 [54,55,56]. Likewise, tumor cells reduce the amount of major histocompatibility complex (MHC) I molecules to evade the interaction with the T cell receptor (TCR). (**5**) HRS cells overexpress molecules such as *PD-L1* and *PD-L2* to inhibit cytotoxic cells binding to PD1.

CD8^+^ T cells in cHL are less abundant than CD4^+^ T cells in cHL tissues and frequently have dysfunctional features [3,9]. Their anti-tumoral activity may be thwarted by the following strategies, summarized in Figure 1:

(1) Secretion of immunosuppressive molecules, such as IL19, TGF-β, PGE2, and Galectin-1, by HRS cells [20,21].
(2) Overexpression of *PD-L1* and *PD-L2* [50] and loss of *MHC-I, MHC-II*, and *CD58* [3] in HRS cells.
(3) Long distance between CD8^+^ T cells and tumor cells [3].
(4) Expression of proteolytic molecules by HRS and mesenchymal stromal cells that cleave NKG2 ligands [20], which are necessary to ensure the cytotoxic function of CD8^+^ T cells [51].

Apart from the activation status of CD8^+^ T cells, their differentiation stages may be relevant in cHL, because CD8^+^ T cell infiltrates are significantly more differentiated in patients than in healthy subjects, with fewer naive and CM CD8^+^ T cells, and more EM [4] and TEMRA CD8^+^ T cells. Moreover, as with CD4^+^ T cells, CD8^+^ T cells can be classified into subsets that produce type 1, 2, and 17 cytokines, termed Tc1, Tc2, and Tc17, respectively. Combining all classification strategies, it was found that cytotoxic T cells express *GrB* and are more differentiated and Tc1-polarized in cHL patients. By contrast, Tc2 cells are less frequent and less differentiated, with a prevalence of CM cells and a lack of EM Tc2 cells [3,30,36]. However, there are no studies that relate Tc1, Tc2, or Tc17 subpopulations to prognosis in cHL, even though they are relevant in other tumors [52].

## 5. Natural Killer Cells

Although NK cells are scarce and fail to kill HRS cells in cHL, their infiltrates and activation status confer a favorable prognosis [10]. Healthy subjects have significantly more NK cells in peripheral blood than cHL patients at all stages of differentiation [29,50]. Advanced disease or adverse prognostic factors exacerbate the NK deficiency [10]. As HRS cells produce cytokines that can attract NK cells [3,20] and harbor inactivating mutations of *β-2-microglobulin* that lead to the loss of *MHC-I* expression, TME NK cells may be expected to be invariably activated [3]. However, their qualitative and quantitative deficiency could be caused by the following strategies, summarized in Figure 1:

(1) The release of soluble factors by HRS cells that dull NK cell activation and recruitment [3] and block the production of some other NK-activating cytokines [21].
(2) A physical barrier of myeloid and T cells around HRS cells that blocks the access of NK cells to tumor cell shores [3].
(3) The help from the TME. As an example, TAM-like monocytes suppress the activation of PD-1^+^ NK cells in vitro, a process that can be reversed by PD-1 blockade [50]. This therapy augments the amount [29] and cytotoxicity [50] of NK cells in the peripheral blood of patients who respond.

Circulating NK cells are customarily distinguished based on their surface expression of CD56 and CD16 molecules. Most of these cells are CD56^dim^ CD16^+^; they are cytotoxic and mediate antibody-dependent cellular cytotoxicity. The other cells are CD56^bright^ CD16^−^, and are only weakly cytotoxic before activation [21,50]. Nevertheless, phenotypic analysis of tissue-resident, peripheral blood, and bone marrow NK cells has provided insight into a broader spectrum of NK cells [10]. This fact, coupled with the plasticity within circulating NK subsets in response to cytokine signals [50], makes the differential role of NK subsets in mediating a regulatory versus cytotoxic function against cancer difficult to study in cHL. To reflect this spectrum of different phenotypes, NK cell subsets can be defined using additional markers (CD57, GrB, CD161, and DNAM-1) [3,29].

It is notable that most NK cells in the peripheral blood of cHL patients are CD56^bright^ CD16^−^ [21]. Of the rest, the new CD56^+^ CD16^+^ PD-1^+^ GrB^−^ and CD56^dim^ DNAM-1^−^ NK cell populations are enriched in cHL patients relative to healthy donors. Both populations present dysfunctional NK features [3,29], suggesting that they help create a tumor-permissive microenvironment. In contrast, high frequencies of CD56^dim^ CD16^bright^ CD57^+^ NK cells in cHL biopsies are associated with good prognostic factors [10].

In addition, a recent cHL study of 43 patients found that the number of NK T (CD3^+^ CD16^+^ CD56^+^) cells increased during treatment [53], suggesting that they may have anti-tumoral effects in the cHL TME. Considered as a whole, these data indicate that only some NK cells kill HRS cells and improve the outcome of the patients, whereas others remain dysfunctional and fail to elicit an anti-tumor response.

## 6. B Cells and Plasma Cells

Non-malignant B cells are relatively abundant in the cHL TME, although their biological contribution is still obscure [21,57]. Many authors have linked B cells, their associated markers (such as CD20 and BCL11A) [2,5,21,58], and their gene signatures [5,59] with favorable outcomes and better overall survival in cHL. Accordingly, cHL patients have significantly fewer B cells than healthy subjects [29]. In contrast, other studies have found no differences in total B cell frequencies, although more specifically defined B cell clusters, such as CD73^+^ memory B cells, were less abundant in cHL patients [30]. The best-known explanation of these findings relies on the competition with malignant cells for growth signals [21]. It is likely that only some B cell subsets contribute to the favorable outcome, but it is also possible that the classic markers include more cell types than just B cells.

Plasma cells originate from the activation of marginal zone B cells by antigens, whose function is to provide high-affinity antibodies during their long life in the bone marrow [60]. Its infiltration in cHL is a variable feature of the disease [5,20]. A recent analysis of the role of CD138^+^ plasma cells in a cohort of 124 Swedish cHL patients revealed that greater plasma cell infiltration is correlated with an advanced stage of the disease and poor survival [57], although the significance of the association was not maintained in the multivariate analysis. It is contrary to other authors’ findings, in which naive IgM-producing plasma cells were associated with better survival [2,61]. As terminally differentiated plasma cells do not express the CD20 marker, and because CD138 is specific to plasma cells that have already undergone affinity maturation [57], cHL TME might prevent plasma cells from terminal differentiation to escape the immune attack. However, Tudor et al. explored different terminally differentiated plasma cells in cHL, and found that CD138^+^, IgG^+^, and IgG4-producing plasma cells did not influence survival [61].

## 7. Monocytes and Macrophages

### 7.1. Monocytes

Monocytes respond to local environmental signals when they extravasate into the tissues and differentiate to macrophages, myeloid-derived suppressor cells (MDSCs) [62], or monocyte-derived DCs (Mo-DCs) [63], although their mechanism of differentiation remains unclear. Alteration of their phenotype causes immune suppression through innate and adaptive immunity, impairing DC differentiation and T cell proliferation [13]. Additionally, they induce angiogenesis and metastasis, explaining why they promote tumorigenesis, and why monocytosis is a poor prognostic factor in many tumors, including B cell lymphomas. Nevertheless, the immunosuppressive activity of the various monocyte subpopulations in lymphomas remains poorly understood [62].

Monocytes are typically divided into classic (CD14^+^) and non-classic (CD14^dim^ CD16^+^) subtypes. Both subsets suppress CD8^+^ T cell growth and IFN-γ production in vitro through different mechanisms. CD14^+^ HLA-DR^low^ monocytes form another subset that produces less IFN-γ and is less capable of generating mature dendritic cells. These three monocyte subgroups are related to tumor development and greater aggressiveness in non-Hodgkin lymphoma (NHL) [13,62]. Recently, another subset of circulating monocytes (CD14^+^ CD163^+^) was proposed, for which pro-tumoral immune effector responses could be relevant in B cell lymphomas [50].

In cHL, the total number of monocytes [12], their gene signatures [5,58], and their associated markers [62,64] are predictors of poor prognosis. Consistent with this, cHL patients have higher frequencies of classical monocytes than do healthy subjects [29]. Furthermore, monocytes can be polarized in vitro to the M2-like immunosuppressive phenotype (defined by *CD163* expression) by HRS cells [12]. As CD68^+^ and CD163^+^ macrophages are usually detected in cHL tissues and their density within tumor samples has been proposed as being a predictor of poor prognosis [40,64], it may be associated with CD14^+^ CD163^+^ monocytes as precursors.

### 7.2. Macrophages

Macrophages derive from hematopoietic progenitors of the bone marrow and are the first line of defense against pathogens. They maintain homeostasis and control inflammation through innate immunity. Initially, they are monocytes in circulation until they differentiate to macrophages under the influence of specific growth factors while infiltrating the hosting tissues, in which they adapt to the local microenvironment [65].

Tumor-infiltrating macrophages, also named tumor-associated macrophages (TAMs), have immunosuppressive roles in B cell lymphoma, blocking effective anti-tumoral immune responses [50]. High levels of macrophages, their associated markers (morphology, *CD68, ALDH1A1, MMP11*, and *LYZ/STAT1* expression), and their gene expression profiles, are recurrently observed in cHL patients associated with poor outcomes [2,5,9,27,58,66], although the *CD68* analysis leads to conflicting results [21,67,68]. The explanation may depend on the expression of *CD68*, which is not restricted solely to macrophages [21], or the fact that intermediate macrophage frequencies produce the best outcomes, superior to those in cases with fewer amounts [69].

TAMs are supposed to polarize either to the M1 subtype, which is thought to promote anti-tumor activity, or to M2, which leads to tumor promotion [15,65,70], suppresses cytotoxic T cell activity, and attracts Tregs [21]. For this reason, the M2 phenotype is associated with tumor progression and worse overall survival in cancer patients [70,71]. However, TAMs are exceptionally plastic and heterogeneous, and their polarization encompasses a continuum that does not fit the oversimplified M1/M2 classification, under either normal or pathological conditions [15,65,72,73]. Consequently, they are better classified as “M1-like” or “M2-like” macrophages [15,74], rather than M1 or M2 macrophages. TAM polarization is controlled by multiple interconnected pathways [15] and some molecules [75].

The microenvironment of cHL is abundant in TAMs, especially in EBV^+^ cases [21]. Their polarization to M1-like or M2-like macrophages is heterogeneous in biopsy samples, indicating distinct biological features of the tumors [58]. Immunohistochemistry (IHC) to detect M1-like cells (CD68^+^ CD86^+^) or M2-like cells (CD163^+^ CD206^+^) has been used to quantify and classify them [76]. Many studies have found that CD68^+^ CD163^+^ M2-like TAMs are associated with poor clinical outcomes in patients with cHL [20,21,36,50], whereas others have not found such an association and have highlighted problems arising from technical heterogeneity [36,69]. It is notable that elevated IL10 levels in pre-treatment serum are associated with decreased progression-free survival (PFS) in cHL patients, a cytokine known to polarize TAMs to the M2-like phenotype [3].

Their extreme plasticity has called the association between TAMs and patient prognosis in cHL into question [15,16,21,58]. Moreover, M1-like macrophages in cHL remain unexplored, probably due to the lack of specific markers [77]. Another problematic finding is that CD163^+^ cells can outnumber CD68^+^ cells in cHL, indicating that *CD163* can be expressed in cells other than macrophages, including DCs [69]. Macrophage subsets in addition to M1-like and M2-like phenotypes have been described. One of them comprises CSF1R^+^ macrophages, which have recently been associated with shorter survival in cHL; this may be because maturation and differentiation of tissue macrophages depend upon interactions between CSF1R and its ligands [36]. Other subsets include the newly identified MYC^+^ CD163^+^ and MYC^+^ CD68^+^ groups, which are associated with worse outcomes in cHL. Accordingly, *MYC* is supposed to control the expression of M2-specific genes in macrophages, and deficiency in MYC^+^ macrophages inhibits tumor growth in mouse models [69].

## 8. Myeloid Cells

Myeloid cells are thought to comprise neutrophils, MDSCs, macrophages, and some monocytes, although some authors include all granulocytes, monocytes, macrophages, and DCs [78]. Tumor-infiltrating myeloid cells are theoretically restricted to either TAMs, Tie2^+^ monocytes, neutrophils, or MDSCs. They control tumor-associated immune suppression, invasion, and metastasis. Myeloid cells are mainly studied by IHC using one or at most two markers, most commonly CD68 or CD163, but without addressing their phenotypic complexity or possible divergent functions in the TME [72].

### 8.1. Myeloid-Derived Suppressor Cells

MDSCs are a heterogeneous population of activated immature myeloid cells that can suppress T cell function. Their origin is under investigation, although they are believed to be hematopoietic progenitor cells generated in the bone marrow that fail to undergo terminal differentiation to mature monocytes or neutrophils before being released into the circulation. In particular, tumor-infiltrating MDSCs are believed to be modified versions of immunosuppressive neutrophils that have adapted to their environment [72].

MDSCs are characterized by the expression of markers commonly associated with granulocytes at different stages of differentiation [21,79], or monocytes [62], although this is controversial. Specifically, human MDSCs are described as lineage-negative cells that co-express *CD11b* and *CD33* but lack *HLA-DR* [72], with an additional division into monocytic (m-MDSCs) and granulocytic (g-MDSCs) subsets. Depending on the source, m-MDSCs are defined as CD14^+^, or CD14^low^, as well as g-MDSCs, which are identified as CD14^−^ or CD15^+^, so caution is needed when interpreting results, drawing conclusions, and comparing studies. MDSCs can also be classified by heterogeneous maturation levels and phenotypes, including immature/undifferentiated CD34^+^ MDSCs [79,80].

In cancer, high frequencies of MDSCs are associated with poor prognosis and tumor progression, including in B cell lymphoma [39,62]. MDSCs have also been identified in the peripheral blood of cHL patients [21], where they are recruited by the release of factors such as indoleamine 2,3-dioxygenase [81]. They seem to be associated with disease aggressiveness and to be prognostically significant [21], although different technical approaches have produced conflicting results [72]. CD34^+^ MDSCs are related to poor outcomes in cHL [79], suggesting that the more immature the MDSCs are, the more they help tumor progression.

The consensus is that g-MDSCs are the low-density, immature, and mature granulocytes (CD66b^+^ CD33^dim^ HLA-DR^−^) recovered from the mononuclear cell fraction after density gradient centrifugation of peripheral blood, and that they are immunosuppressively active [80]. This is the only method to collect g-MDSCs, whose immunophenotype does not differ from mature neutrophils. Some studies of g-MDSCs in cHL describing them as a CD14^−^ population in peripheral blood have found an association with unfavorable prognosis [53,79], as well as others representing them as CD14^−^ CD15^+^ plus the common MDSC markers [82]. Not surprisingly, g-MDSCs were found to be mostly composed of mature CD11b^+^ CD16^+^ low-density neutrophils in an activated state [80].

m-MDSCs form an important subset of MDSCs in B cell lymphomas [62] because they block effective immune responses [50]. Although CD14^+^ m-MDSCs are correlated with more aggressive disease, suppressed adaptive immunity, and a greater propensity to relapse post-therapy in NHL [62,80], cHL m-MDSCs have yet to attract much attention from researchers.

### 8.2. Neutrophils

Neutrophils are the first line of defense against invading pathogens. In peripheral blood, they are almost indistinguishable from tumor-resident MDSCs, because both cell subsets are CD33^+^ CD11b^+^ HLA-DR^low^ Arg1^+^ [72]. Therefore, it has been suggested that some MDSC subtypes are derived from the pathological activation of neutrophils, driven by microenvironmental signals [21]. Indeed, neutrophils, identified by their peculiar morphology, are frequent in tumor infiltrates [72], where they are known as tumor-associated neutrophils (TANs) and interact with tumor cells to promote angiogenesis, cancer cell invasion, and metastasis [21].

The idea that neutrophils are involved in cancers such as DLBCL is controversial [62], although their absolute frequency has been reported to be associated with poor prognosis [21,83] and tumor recurrence [72] in cHL compared to healthy donors [29]. One possible explanation for the controversy about whether neutrophils promote or inhibit tumor growth is that, similar to the macrophage paradigm, neutrophils are proposed to have anti-tumor (N1) and pro-tumor (N2) phenotypes in mice that can only be distinguished based on their function [72]. This suggests that the discrepancies in the results could be a consequence of the distinct phenotypes that neutrophils can acquire, as happens with macrophages and other cell types.

Many authors have suggested targeting MDSCs and reprogramming the TAN phenotype to treat cHL refractory patients [21], which has prompted some preclinical studies. Conversely, few clinical trials have been conducted thus far, due to the difficulty of suppressing their action in their role in defending against infections and the risk of induced immunosuppression.

## 9. Mast Cells

Mast cells differentiate from CD34^+^ CD117^+^ pluripotent hematopoietic stem cells on the bone marrow as soon as they complete their maturation in tissues [84]. Although they are ubiquitous and usually present in low numbers, they become more abundant under a variety of disease conditions, including cancer. Mast cells release tumor-promoting cytokines, such as IL10 and TGF-β1, and pro-angiogenic factors and proteases that enhance fibroblast proliferation, suggesting their importance in favoring tumorigenesis [85].

In cHL, mast cells are present at high frequencies [21], promoting fibrosis and HRS cell growth [20], while mast-attracting factors such as CCL5 are released by tumor cells simultaneously [5,12]. Although some studies could not establish a clear correlation [85,86], mast cell infiltration has been linked to poor prognosis in cHL [5,12,20,21], especially in the mixed cellularity (MC) cHL subtype. Interestingly, cHL mast cell frequencies are inversely correlated with those of CD68^+^ and CD163^+^ macrophages and GrB^+^ cytotoxic cells [85].

## 10. Eosinophils

As occurs with mast cells, eosinophils differentiate from CD34^+^ CD117^+^ pluripotent hematopoietic stem cells in the bone marrow when they complete their maturation in tissues. They sense the environment in order to respond with biochemical programs of inflammation or repair and modulate many innate and adaptive immune cells. Eosinophils are a source of anti-tumorigenic and pro-tumorigenic molecules, depending on the milieu [87].

Eosinophils are common in cHL, usually being more abundant in the MC subtype. *IL5, IL9, CCL5, GM-CSF*, and *CCL28* expression by HRS cells helps recruit them to the affected lymph nodes, while eosinophils can enhance the proliferation of tumor cells by different mechanisms, such as CD30L secretion [5,12,20]. Accordingly, many studies have found a correlation between the abundance of eosinophils and inferior prognosis in cHL [5,12,21,88], although evaluation of tissue eosinophilia remains contentious [5,86]. This might be due to the lack of bona fide IHC markers for identifying eosinophils in tissue and their semi-quantitative estimation by morphology in classic reports [11,89].

## 11. Dendritic Cells

DCs initiate and direct immune responses through antigen presentation [17]. They have customarily been defined as antigen-presenting cells that lack other leukocyte lineage markers and express high levels of *MHC-II* (*HLA-DR*) molecules [90]. The common DC progenitor gives rise to either plasmacytoid (p) DCs (CD11c^−^) or one of two myeloid (m) DC (CD11c^+^) subsets, named “mDC1” or “mDC2” [17,91]. These groups shape the adaptive immunity to intracellular and extracellular pathogens, respectively [17]. Usually, DCs are present at low quantities in tissues with difficult access. This, coupled with the lack of distinctive markers for each DC subset, has made it difficult to study them [92].

Although some in silico [8] and in vivo analyses have shown a positive association between the proportion of DCs and outcome in many tumors, the findings in cHL are ambiguous. Recently, it was seen that, when co-cultured, DCs inhibit the proliferation of some cHL cell lines [64]. Additionally, Galati et al. found fewer DCs from all subsets in cHL patients than in healthy controls, but more of them at the end of the treatment than at diagnosis. However, no significant difference in outcome was observed between patients with divergent mDC or pDC levels [91].

### 11.1. Myeloid DCs

Myeloid or conventional DCs consist of CD1c^+^ (mDC1) or CD141^+^ (mDC2) subsets [91]. mDC1 cells promote Th17 responses, produce high levels of IL12, induce and mediate Th1 responses, and can cross-present some antigens to CD8^+^ T cells [17,92], whereas mDC2 cells mediate both Th2 and Th17 responses [17]. Large numbers of CD83^+^ mDCs, which identify a mature subset of mDCs, are associated with improved outcomes in cHL. Remarkably, they are the main DC population, outnumbering the CD123^+^ pDCs. Additionally, supernatants of cHL cell lines induce mDC maturation, possibly through the TNF-α released by HRS cells [64]. Recently, fewer cells of both mDC subtypes were found in patients with advanced-stage cHL, although this was not associated with their outcome [91]. This suggests some degree of depletion of mDCs during cHL progression.

### 11.2. Plasmacytoid DCs

pDCs is a subset renowned for its anti-viral activity and type-I IFN production [17]. It comprises CD123^+^ and CD303^+^ cells [91]. CD123^+^ pDCs are the most abundant DC type in cHL [64]. Associated pDC biomarkers such as BCL11A are related to favorable outcomes in some cHL studies [5], whereas other findings using gene expression profiling are counter to this [64]. Nevertheless, they agree about the lack of pDCs in cHL patients relative to healthy subjects [91]. Additionally, the pDCs of cHL patients produce little TNF-α relative to the amounts seen in healthy donors [93], suggesting that pDCs in the cHL TME are switched to a more dysfunctional phenotype.

The role of pDCs in cHL needs to be explored further to explain the mixed results obtained so far. These discrepancies may have arisen because the samples were too small, or because there are more pDC variants, with different abilities to induce T cell proliferation and to produce cytokines [91]. In addition, CD123 is used as a specific marker of pDCs, but it must be remembered that HRS cells also express it in 90% of cHL cases [94], especially in NS cHL, and overexpress IL3 receptors [95]. Other data suggest that *CD123* expression also occurs in M2-like macrophages in the TME of cHL [96]. Thus, the findings ascribed to cells labeled “pDCs” may actually arise from more cell types.

### 11.3. Follicular DCs (fDCs)

In cHL, the absence of CD21^+^ fDCs is associated with unfavorable outcomes [5], and high frequencies of CD21^+^ cells are correlated with favorable outcomes [61]. However, it is not clear whether CD20^+^ B and fDC cells present in the TME are residues of the original lymphoid tissue architecture or actively formed new follicular structures in the tumor.

## 12. Discussion and Perspectives: Functional Signatures of the Tumor Microenvironment

Around 30% of patients with cHL in advanced stages or atypical cases that present with limited stages have primary refractory tumors that cannot be cured of the disease. Currently, these refractory patients are candidates for salvage therapies by a combination of bone marrow transplant and intensive chemotherapy. Additionally, brentuximab vedotin and the checkpoint inhibitors have substantially affected second-line therapy and post-transplant management of the relapsed and refractory cHL. However, although many patients may achieve prolonged disease control with checkpoint inhibitors, the majority eventually progress and require additional therapy.

This overview of the cHL microenvironment highlights a preponderance of immunosuppressive cell populations (Tregs, Th2 cells, M2-like macrophages, and MDSCs) with ineffective cytotoxic cells (CD8^+^ T cells, Th1 cells, and NK cells) [21,29]. Apart from the direct crosstalk between tumor cells and TME cells, the topography of the tumor cells, their spatial organization, and their interactions might explain how HRS cells escape cytotoxic attack. Specifically, the most immunosuppressive subpopulations surround nearby tumor cells [27]. In contrast, CD8^+^ T cells, Th17 subsets, NK cells, and PD-L1^−^ CD86^low^ TAMs, which should have residual anti-tumoral cytolytic activity, occupy regions at a distance from HRS cells [3].

Table 1 summarizes the cell populations that have the most important influence on tumor growth and therapeutic response. There are newly identified cell populations that cannot be ascribed to any known cell type, although they are relevant to cHL pathogenesis. For instance, patients in the remission phase and at relapse have more IFN-γ^+^ IL4^+^ cells and fewer IFN-γ^−^ IL4^−^ CD4^+^ T cells than newly diagnosed patients. None of these cell groups correspond to Th1, Th2, or any known Th cell subtype. However, the frequency of those cell clusters is not associated with the cHL outcome [33]. Another example of an unclassified subset is the CD3^−^ CD68^+^ CD4^+^ GrB^+^ cell population, which is higher in cHL patients who respond to anti-PD-L1 therapy and in healthy donors than in newly diagnosed patients [29].

The recent clinical success of immunotherapy in cHL represents a milestone in the management of chemorefractory patients and, probably, also for first line strategies. It confirms the major genetic dependencies of the neoplastic HRS clones and proves the relevance of the TME. Targeting the PD-1/PD-L1 pathway can significantly improve PFS in 86% of patients with relapsed or treated refractory HL [97]. However, the response rate to immunotherapy is still limited and reliable biomarkers need to be identified [29]. Most of the elements from the TME previously reported to be associated with clinical responses were identified in a conventional chemotherapy setting and correspond to discrete cell populations that are probably unrelated to the mechanisms of immune checkpoint inhibitors.

It remains unclear why the immunotherapeutic resistance still exists, or develops after treatment [21]. Major limitations of the research were apparent from the scarcity of observations in longitudinal tumor samples from patients treated in the setting of well-designed clinical trials [14]. For example, Cader et al. recently described the dependence on more complex functional signatures and identified significant roles of previously unrecognized cell phenotypes [29].

CTLA4^+^ is another important cell subset. It is more frequent in the HRS proximal region and comprises both Treg and non-Treg populations. Surprisingly, they outnumber PD1^+^ and LAG3^+^ T cells. T cells rosetting HRS cells are more frequently CTLA4^+^ than PD-1^+^ or LAG3^+^. Additionally, the density of CD8^+^ CTLA4^+^ cells is higher in cHL cases than in RLNs. HRS cells and some subsets of TAMs are positive for the CTLA4 ligand CD86, supporting the idea that it is worthwhile targeting the CTLA4-CD86 axis to treat patients of cHL who relapse after anti-PD1 therapy [3,98].

**Figure 2:**
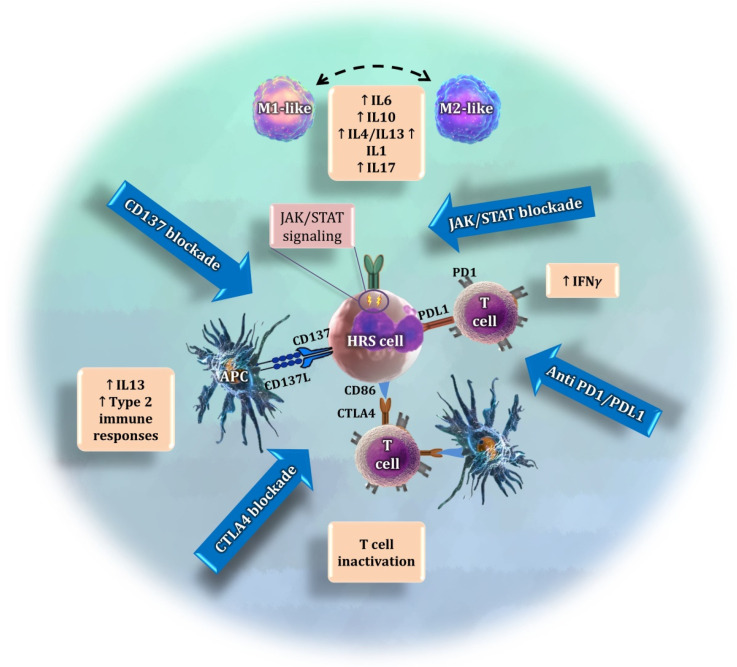
Summary of four different approaches to target the interaction of the tumor microenvironment with HRS cells. The JAK/STAT pathway is overactivated in tumor cells, causing the release of cytokines that regulate the polarization of macrophages [105,106,107] and other immunosuppressive activities. The current immunotherapy approaches target the interaction between *PD-L1*, which is overexpressed by HRS cells, and PD1. CTLA4 is another promising immune checkpoint whose blockade may exhibit anti-tumoral effects. Finally, *CD137* is expressed by tumor cells as an additional immune scape mechanism. Its blockade may avoid the interaction with CD137L and prevent the release of tumor-promoting cytokines.

Other approaches for targeting the TME in cHL are currently under investigation (Figure 2). A promising target is represented by the JAK/STAT signaling pathway, whose overactivation is a common feature of cHL, along with NF-kB activation [99,100]. Constitutive activation of the pathway may be the consequence of *JAK2* activation/overexpression secondary to 9p24.1 genomic amplification, activating mutations in molecules such as *STAT3* and *STAT6*, or inactivating mutations and deletions in *SOCS1*-negative or *PTPN1*-negative regulators [101,102,103]. In addition, around 30% of the patients are infected by EBV, which also contributes to the constitutive activation of the JAK/STAT pathway [104]. This persistent activation of the JAK/STAT signaling pathway is also related to the suppression of the anti-tumor immunity and the tumor-promoting inflammation that characterizes the cHL TME [104]. The pharmacological blockade of JAK/STAT has demonstrated some activity in preliminary clinical trials in advanced cHL, but is of limited efficacy as monotherapy [99,100]. The potential synergies of anti-JAK/STAT therapy in combination with immune checkpoint inhibitors or standard chemotherapy agents requires clinical validation. In addition, little is known about the complex biological consequences of this blockade.

It has recently been shown that HRS cells ectopically express the cytokine receptor CD137 (*TNFRSF9*) as an additional immune escape mechanism. Two independent studies have found that, in 86% of cHL cases, HRS cells stained positive for CD137 [108,109]. This was a surprising finding, since HRS cells are derived from germinal center B cells, in which CD137 could not be detected [108,109]. Ectopic *CD137* expression on HRS cells enables the secretion of IL13, which directs the immune response towards a type 2 response, leading to a further reduction in IFN-γ [110]. A novel therapeutic strategy could seek to neutralize CD137 in HRS cells. This may not only prevent the downregulation of CD137L on antigen-presenting cells (APCs), which is pivotal for inducing a type 1 immune response, but could also prevent an IL13-driven immune escape [110].

**Figure 3:**
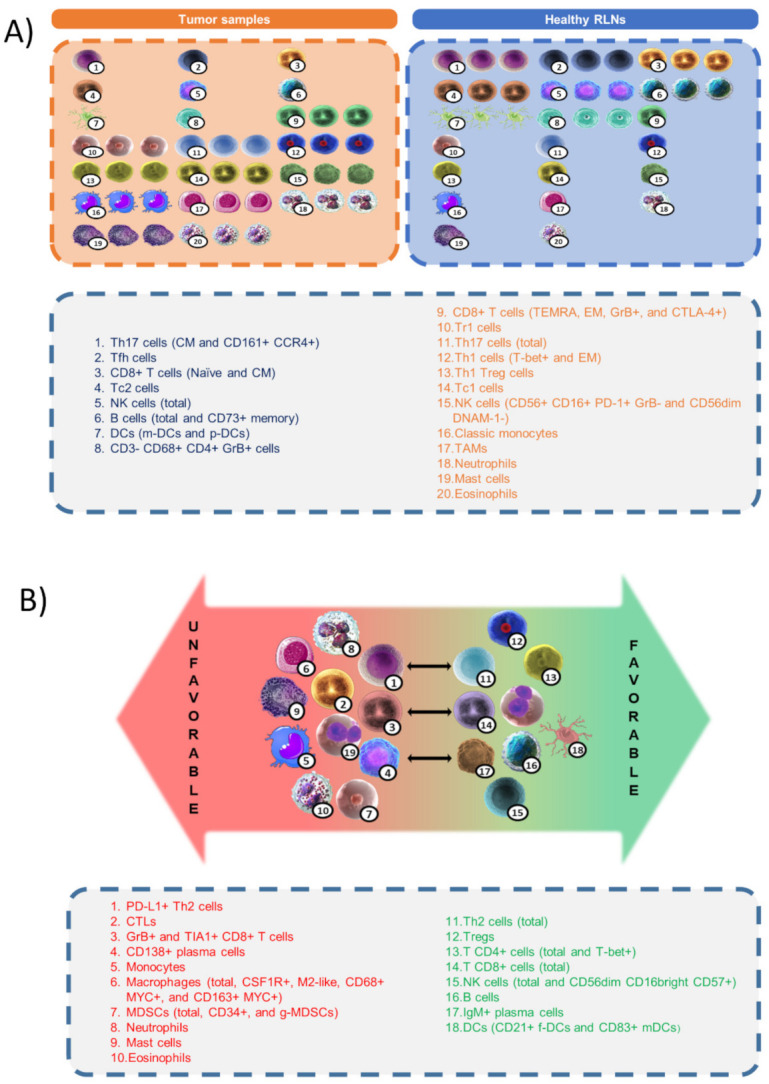
(**A**) Differences in amounts of cell populations between healthy reactive lymph nodes (RLNs) and classic Hodgkin lymphoma (cHL) samples. (**B**) Classification of cell types and immune signatures with respect to favorable or unfavorable outcomes in cHL patients.

The TME is a complex network that comprises cellular and non-cellular components, induced and shaped by the HRS clone through abnormal cytokine production because of genetic alterations, that, in turn, might be the consequence of clonal selection by the attracted immune cells (Figure 3). Current differences in amounts of many cell types in the TME comparing tumor and healthy tissues are qualitative and not quantitative, because they are obtained by different methods in different research groups and differences cannot be compared. The differences between “favorable” and “unfavorable” phenotypes based on the clinical outcome of the patients is a matter of the final balance between the environmental and genetic alterations that modify homeostatic immunity, the influence of the host’s idiosyncrasies, and the different therapeutic regimens.

## 13. Conclusions

Reliable biomarkers for current therapies need to be identified as part of more rational functional signatures. These should consist of complex descriptors that integrate:

(1) The individual genetic lesions of the neoplastic clone (such as the 9p24.1 amplification, or JAK/STAT and NF-kB pathway overactivation due to gene mutations [111,112,113,114,115]).
(2) Tumor descriptors, such as gene expression analyses based on pathway-related genes, that could be upregulated or suppressed in the responsive versus non-responsive TME.
(3) Phenotype-specific dysregulated descriptors of the chemokine milieu.
(4) Relevant cell populations and functionalities (such as T cell and monocyte signatures).

Two major limitations must be overcome to enable complex functional signatures of the TME to be characterized: (1) the validation of robust technologies for in situ analyses of complex phenotypes such as multiplex immunofluorescence techniques, immune-related gene expression profiling, and single-cell RNA sequencing; and (2) the development of appropriate experimental models for cHL, as multicellular organoids. The characterization of functional signatures will eventually provide insights into major mechanisms of resistance to immunotherapy in cHL and accurately classify tumors into being immunotherapy responsive and inflamed non-responsive. This approach could ultimately identify potential synergistic drugs capable of converting an unfavorable microenvironment phenotype into a more favorable one, aiding current immunotherapy and guiding rational therapeutic combinations.

## Figures and Tables

**Table 1:** Summary of cell populations that influence the therapeutic response in classic Hodgkin lymphoma. They are classified in terms of their association with favorable or unfavorable outcomes in cHL patients.

Cell Population			Outcome Association	Details	References
T CD4^+^ cells		General	Favorable	Better outcome	[23,27,29]
		T-bet^+^	Favorable	Better outcome and response to anti-PD-L1 immunotherapy	[23,27,29]
	Th2	General	Favorable	Improved disease-free and event-free survival	[35]
		PD-L1^+^	Unfavorable	Worse outcome and shorter survival	[36]
	Tregs	CD25^+^ and FOXP3^+^	Favorable	Positively associated with survival rate	[2,5,23,37,42]
		FOXP3^+^ GrB^+^	Favorable	Improved survival	[5,14]
T CD8^+^ cells		General	Favorable	Better outcome, particularly in the advanced-disease group	[9,27]
		Cytotoxic (CTLs)	Unfavorable	Worse clinical outcome	[5,7,14,36]
		GrB^+^ TIA1^+^	Unfavorable	Worse prognosis	[5,7,14,36]
NK cells		General	Favorable	Infiltration and activation confer better prognosis	[10]
		CD56^dim^ CD16^bright^ CD57^+^	Favorable	Better prognostic factors	[10]
B cells		General	Favorable	Better outcome	[2,5,21,58]
		CD138^+^ Plasma cells	Unfavorable	Associated with advanced stage and poor survival	[57]
		IgM^+^ Plasma cells	Favorable	Better survival	[2,61]
Monocytes		General	Unfavorable	Poor prognosis for frequency, gene signature, and markers	[5,12,28,58,62]
Macrophages		General	Unfavorable	Poor outcomes Intermediate numbers show best outcomes	[2,5,9,27,58,66] [69]
		CSF1R^+^	Unfavorable	Shorter survival	[36]
		M2-like (CD68^+^ CD163^+^)	Unfavorable	Poor clinical outcomes	[20,21,36,50]
		CD68^+^/CD163^+^ MYC^+^	Unfavorable	Worse outcome	[69]
MDSCs (CD11b^+^ CD33^+^ HL-DR^−^)		General	Unfavorable	Correlated with disease aggressiveness and poor prognosis	[21]
		CD34^+^	Unfavorable	Poor outcomes	[79]
		g-MDSCs (CD14^−^ or CD66^+^ CD33^dim^, or CD14^−^ CD15^+^)	Unfavorable	Worse prognosis	[53,79,82]
Neutrophils		General	Unfavorable	Poor prognosis and tumor recurrence	[21,72,83]
Mast cells		General	Unfavorable	Poor prognosis and fibrosis promotion	[5,12,20,21]
Eosinophils		General	Unfavorable	Inferior prognosis	[5,12,21,88]
Dendritic cells		CD21^+^ Follicular DCs	Favorable	Better outcome	[5,61]
		CD83^+^ mDCs	Favorable	Improved outcome	[64]

## Data Availability

Data sharing is not applicable to this article, as no new data were created or analyzed in this study.
